# Escinosomes: Safe and Successful Nanovesicles to Deliver Andrographolide by a Subcutaneous Route in a Mice Model of Oxaliplatin-Induced Neuropathy

**DOI:** 10.3390/pharmaceutics14030493

**Published:** 2022-02-24

**Authors:** Giulia Vanti, Michela Capizzi, Lorenzo Di Cesare Mannelli, Elena Lucarini, Maria Camilla Bergonzi, Carla Ghelardini, Anna Rita Bilia

**Affiliations:** 1 Department of Chemistry, University of Florence, Via Ugo Schiff 6, 50019 Florence, Italy; giulia.vanti@unifi.it (G.V.); michela.capizzi@stud.unifi.it (M.C.); mc.bergonzi@unifi.it (M.C.B.); 2 Pharmacology and Toxicology Section, Department of Neuroscience, Psychology, Drug Research and Child Health (NEUROFARBA), University of Florence, Viale G. Pieraccini 6, 50139 Florence, Italy; lorenzo.mannelli@unifi.it (L.D.C.M.); elena.lucarini@unifi.it (E.L.); carla.ghelardini@unifi.it (C.G.)

**Keywords:** andrographolide, escin, escinosomes, nanovesicles, oxaliplatin-induced neuropathy, subcutaneous administration

## Abstract

Andrographolide (AG) is a natural diterpene lactone endowed with considerable therapeutic potential for treating numerous diseases, including neurological disorders, but its low aqueous solubility and scarce bioavailability limit its clinical use. To overcome this problem, AG was encapsulated in escinosomes, special nanovesicles made of escin (ESN), a natural saponin, and phosphatidylcholine. Escinosomes loaded with AG had an average size of 164.7 ± 13.30 nm, optimal polydispersity index (0.190 ± 0.0890) and high ζ-potential (−35.4 ± 0.451 mV), and significantly loaded the active substance—the encapsulation efficiency of AG was about 88%. Escinosomes allowed the prolonged release of AG over time, without burst effects—about 85% AG was released after 24 h. Morphological analysis by cryo-transmission electron microscopy showed nanovesicles with a spherical shape, unilamellar and oligolamellar structures, and dimensions in agreement with those measured by dynamic light scattering. In addition, stability studies were performed on AG-loaded escinosomes stored for one month at 4 °C. The pain-relieving efficacy of these nanovesicles was tested in a rat model of oxaliplatin-induced neuropathy. AG-loaded escinosomes, subcutaneously administered, effectively reduced the thermal allodynia characteristic of chemotherapy-induced neuropathy, enhancing and prolonging the effect of the natural compound. Overall, AG-loaded escinosomes were found to be excellent for loading AG, physically and chemically stable for one-month storage, and with controlled-release properties, making the formulation an ideal pharmacological approach for persistent pain treatment.

## 1. Introduction

Neuropathy is a common, dose-dependent adverse effect of the treatment with oxaliplatin, a third-generation platinum-based anticancer agent, mainly used in the therapy of colorectal cancer. The development of neuropathy leads to a detrimental dose reduction and discontinuation of the cancer therapy, with severe implications for patients’ health [1]. Patients affected by neuropathy experience tingling, pinprick and loss of sensation in the extremities, often amplified by contact with cold objects. These neurological complications can largely persist after the end of the chemotherapy [2]. Currently, antidepressants and antiepileptics are considered the elective therapy for these types of pain, but they show a low efficacy with several adverse side effects [3]. It is thus clear that the research of effective and safe treatments for this type of persistent pain be strongly pursued.

**Figure 1:**
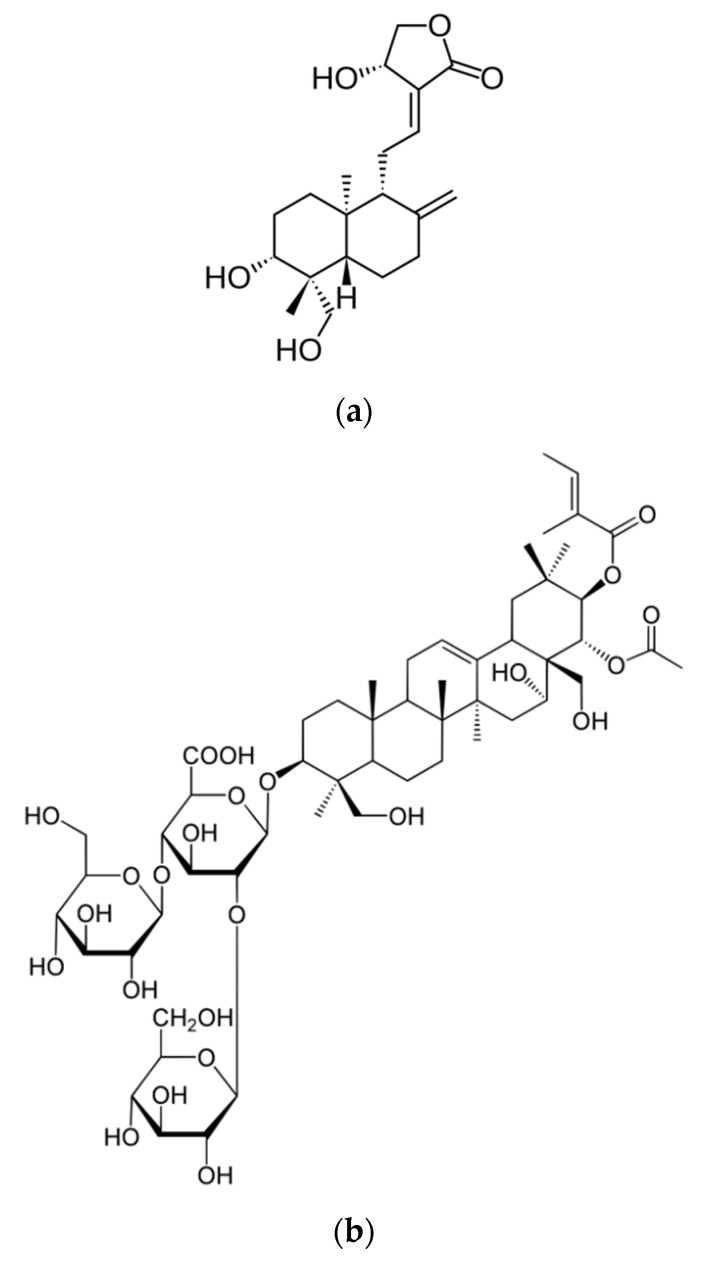
Structural formula of (**a**) andrographolide and (**b**) escin.

Andrographolide (AG) (Figure 1a) is a natural product with various potential therapeutic benefits for the broad range of multifunctional properties, principally antioxidant, neuroprotective, and anti-inflammatory. However, AG has not reached its therapeutic potential due to its unsuitable drug like biopharmaceutical properties [4,5]. Indeed, AG has poor water solubility and a short biological half-life (t_½_ = 1.33 h) after a single oral dose. It is unstable in gastrointestinal media, profoundly limiting its distribution and accumulation in the body after administration, resulting in low bioavailability [6,7].

Consequently, the production of suitable delivery systems for this natural compound can pave the way to new efficacious therapeutic approaches for oxaliplatin-induced neuropathic pain [8,9]. In addition, the subcutaneous administration route was selected in this study to obtain a rapid systemic effect avoiding gastrointestinal transit.

In this study, AG was formulated in escinosomes, new nanovesicles made of phosphatidylcholine and escin (ESN) (Figure 1b), a bioactive triterpene saponin isolated from the *Aesculus hippocastanum*. ESN demonstrated imparting favorable properties to the vesicles, being a bilayer-forming material as previously described [10]. That study reported the investigation of technological properties of three different vesicles: conventional liposomes made of phosphatidylcholine and cholesterol, conventional liposomes plus escin, and vesicles only made of phosphatidylcholine and escin, called escinosomes. Specifically, size, polydispersity index (PdI), deformability, encapsulation efficiency, drug release, and permeability were evaluated and compared. The choice of ESN was based on its amphiphilic chemical structure and numerous pharmacological activities, including anti-inflammatory, anti-edematous and venotonic properties, which can impart a potential biological activity to escinosomes, with a consequent enhanced therapeutic effect. The study demonstrated superior technological characteristics for the newly developed escinosomes, which also preserved the anti-hyaluronidase activity of the saponin [10]. Accordingly, AG-loaded escinosomes were prepared, improved in size and PdI, and characterized with regard to the encapsulation efficiency and release profile of the active substances and chemical/physical stability. In addition, the subcutaneous administration route was selected to obtain a rapid systemic effect avoiding gastrointestinal transit, and the efficacy of acute subcutaneous administration was evaluated in a mouse model of oxaliplatin-induced neuropathy using the cold plate test.

## 2. Materials and Methods

### 2.1. Materials

Andrographolide (98%, AG), escin (95%, ESN), cholesterol, phosphate buffer saline (PBS; 0.138 M NaCl and 0.0027 M KCl; pH 7.4), and all the organic solvents (dichloromethane, methanol HPLC grade and formic acid HPLC grade) were purchased from Sigma Aldrich (Milan, Italy). Soybean phosphatidylcholine Phospholipon^®^ 90G (P90G) was purchased from Lipoid GmbH (Ludwigshafen, Germany) with the support of the Italian agency AVG Srl. Ultrapure water was produced by a Simplicity^®^ UV water purification system provided by Merck Life Sciences Srl (Milan, Italy).

### 2.2. HPLC-DAD Analytical Method

**Figure 2:**
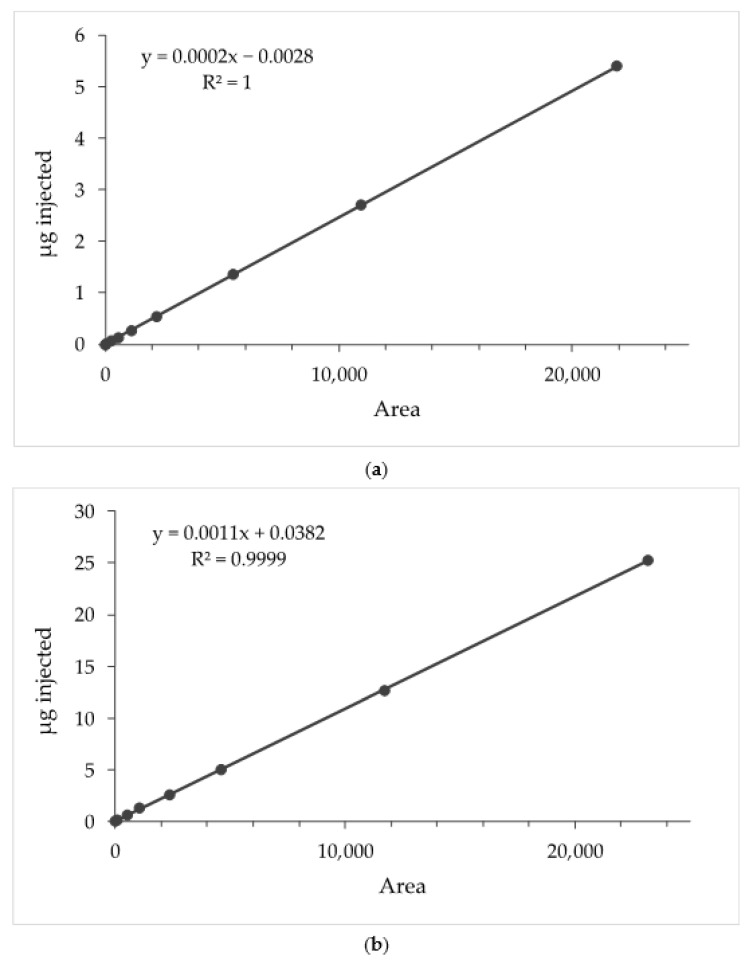
Calibration curves of (**a**) andrographolide and (**b**) escin.

The quantitative analyses of AG and ESN were performed using an 1100 high-performance liquid chromatograph (HPLC) coupled with a UV-visible detector (DAD) by Agilent Technologies Italia Spa (Rome, Italy) and an Eclipse XDB-C18 column (150 × 4.6 mm, 3.5 μm; Agilent Technologies Italia Spa (Rome, Italy), working at 25 °C. Elution was carried out using (A) acetonitrile and (B) water (pH 3.2 by formic acid) as mobile phase, at a flow rate of 0.5 mL/min, and with the following multistep linear gradient: 0.1–2.0 min 30% (A), 2–20 min 30–99% (A), 30–35 min 99–30% (A). The post-time was 5 min. Chromatograms were recorded at 226 nm (AG) and 210 nm (ESN). The calibration curves were obtained by dissolving AG and ESN in methanol at 0.5 mg/mL and 5 mg/mL, respectively (Figure 2a,b). A linear relationship was obtained for both molecules. The coefficient of determination (R^2^) was 0.99999 for AG in the range 5.00–0.01 μg. R^2^ was 0.99997 for ESN in the range 25.0–0.1 μg.

### 2.3. Preparation of Escinosomes

Escinosomes were prepared by the thin layer evaporation method adding ESN (empty escinosomes) or ESN plus AG (AG-loaded escinosomes) to the organic phase [10,11]. Briefly, 330 mg of P90G, 50 mg ESN, and 20 mg AG were dissolved in dichloromethane and methanol in a 1:1 volumetric ratio using an ultrasonic bath for 1 min. The molar ratio P90G:ESN:AG was 23.8:1:1.3. The organic solvents were evaporated with a rotavapor under reduced pressure for 20 min at 30 °C. The obtained lipid film was successively hydrated with 10 mL of water using mechanical stirring for 30 min at 330 rpm and maintaining the flask in a water bath at 35 °C. Then, the Sonopuls Ultrasonic Homogenizer HD 2200 by Bandelin electronic GmbH & Co. KG (Berlin, Germany) coupled with the MS 72 probe was used to reduce vesicle size and improve sample homogeneity. Specifically, 10 min of sonication was applied with 1/2 s on and 1/2 s off cycles at 48% power, keeping the sample immersed in ice to avoid excessive heating and degradation of the lipids. Successively, the formulation was let to equilibrate at 25 ± 2 °C under magnetic stirring for 15 min and then in the fridge at 4 °C for one night.

### 2.4. Physical Characterization of Escinosomes

#### 2.4.1. Dynamic and Electrophoretic Light Scattering

The formulation was analyzed during the preparation and optimization steps by Dynamic and Electrophoretic Light Scattering (DLS and ELS) using a Zetasizer Nanoseries ZS 90 equipped with a 4 mW He-Ne laser, operating at 632.8 nm, and an APD detector provided by Malvern Panalytical Ltd. (Worcestershire, UK) [12]. Measurements were performed in triplicate with a scattering angle of 90° at 25 °C. Dimensions (average hydrodynamic diameter, size; nm), homogeneity (polydispersity index, PdI; dimensionless parameter), and ζ-potential (mV) of the escinosomes were determined by 50-fold dilution of the formulation with ultrapure water. All data were analyzed by the cumulants method.

#### 2.4.2. Cryo Transmission Electron Microscopy

The morphology of the developed nanovesicles was evaluated using a Thermo Scientific™ Glacios™ cryo transmission electron microscope (Cryo-TEM) by FEI (Hillsboro, OR, USA), equipped with a highly sensitive TEM direct electron detector (DED) Falcon III by FEI (Hillsboro, OR, USA) at an accelerating voltage of 200 kV. The low-dose mode in EPU software (FEI (Hillsboro, OR, USA) was used to minimize radiation damage during image acquisition. Images were obtained with Falcon III DED at 57,000× magnification with a defocus value of −4 μm. The accumulated total dose per image did not exceed ~26e^−^/Å^2^. Holey carbon EM grids (Quantifoil R2/2 Cu300) were glow-discharged (45 s, 20 mA) in the Pelco EasiGlow system. A sample aliquot (3 μL) was applied on the carbon side of the EM grid, inside a chamber at 20 °C and 100% humidity. Once the sample was deposited, the grid was blotted on both sides for 1.0 s and plunge-frozen into precooled liquid ethane. This step reduces the sample amount on the grid to create an amorphous ice film with a thickness of 50–200 nm where the molecules are fixed. This procedure was performed using a Thermo Scientific™ Vitrobot Mark IV by FEI (Hillsboro, OR, USA), allowing blotting and instant sample freezing by rapid immersion in liquid ethane (melting temperature of 90.3 K). The procedure results in embedding the samples in a thin layer of amorphous ice to preserve them in their native state and protect them from radiation damage. Instant freezing in liquid ethane is necessary to avoid the formation of structured ice (cubic, hexagonal, or others), compromising the sample architecture and the image quality. The process of sample freezing is called vitrification. The grid was kept in liquid nitrogen for the following steps and then loaded into the microscope. Shape, aggregation principles, size, and other morphological aspects were identified by mapping the grid and observation. Samples were analyzed after a 4-fold dilution in ultrapure water.

### 2.5. Chemical Characterization of Escinosomes

Chemical characterization of escinosomes was carried out by determining the encapsulation efficiency (EE%) and total recovery (R%) of AG and ESN. In order to measure EE%, the not encapsulated drug was removed by the dialysis bag method, performed using Spectra/Por^®^ regenerated cellulose membranes, with 12–14 kDa molecular weight cut-off, by Repligen Europe B.V. (Breda, The Netherlands) [13]. The dialysis bag, filled with 1 mL of formulation, was immersed in 1 L of ultrapure water at 25 ± 1 °C and kept under magnetic stirring at 100 rpm for 1 h. Then, the purified formulation was diluted 20 times in methanol to break up the escinosomes and solubilize the active substances. The samples were vortexed for 30 s, sonicated in an ultrasonic bath for 10 min, and centrifuged at 14,000 rpm for 10 min. The supernatants were analyzed by HPLC-DAD. In parallel, R% was settled using the same method without dialysis and analyzing samples by HPLC-DAD. EE% and R% were calculated as percentages of the concentrations determined by HPLC-DAD analysis, divided by the initial drug concentration determined by weighing, as previously reported [14].

### 2.6. Release Study of ESN and AG from Escinosomes

#### 2.6.1. Selection of Acceptor Medium

Solubility tests of ESN and AG in PBS, 1% *v*/*v* Tween 20/PBS and 5% *v*/*v* Tween 20/PBS, were carried out to select the acceptor medium for the release study [12]. Excess of ESN or AG was added to an exact volume of solution, and the suspension was kept under magnetic stirring for 24 h at 25 ± 1 °C. Then, the samples were centrifuged at 14,000 rpm for 10 min, and the supernatants were analyzed by HPLC-DAD after the necessary dilutions in methanol.

#### 2.6.2. Drug Release Experiment

The dialysis bag method was employed to assess the release rate of ESN and AG from the escinosomes [15,16]. The formulation (2 mL) was placed inside the Spectra/Por^®^ dialysis tubing of regenerated cellulose with molecular weight cut-off of 12–14 kDa (Repligen Europe BV; Breda, The Netherlands) and selective permeability, while the bag was maintained under magnetic stirring at 100 rpm for 24 h in the selected acceptor medium (200 mL), heated at the controlled temperature of 37 ± 1 °C. The acceptor solution (0.5 mL) was collected and replaced by an equal volume of the fresh medium after 30 min and every hour until 7 h. The last withdrawal was done after 24 h. All samples were analyzed by HPLC-DAD.

### 2.7. Stability Studies

Stability studies were carried out by storing the samples at 4 °C in the dark. The physical and chemical parameters of the formulation were monitored for 1 month. Particularly, size, PdI and ζ-potential were measured by DLS and ELS every week, while EE% and R% of ESN and AG were determined by HPLC-DAD after 2 and 4 weeks.

### 2.8. Animals

For the in vivo experiments, Sprague Dawley rats (220–250 g) provided by Envigo (Varese, Italy) were used and housed at 23 ± 1 °C with a 12 h light/dark cycle (light at 7 a.m.), at Centro Stabulazione Animali da Laboratorio (CeSal, University of Florence), in 26 × 41 cm^2^ cages (4 rats in each cage). Rats were fed with a standard laboratory diet and tap water ad libitum. All animal manipulations were carried out according to the Directive 2010/63/EU of the European Parliament and the European Union council (22 September 2010). The ethical policy of the University of Florence complies with the Guide for the Care and Use of Laboratory Animals of the US National Institutes of Health (NIH Publication No. 85–23, revised 1996; University of Florence assurance number: A5278–01). The experiments described were approved by the Italian Ministry of Health and the Animal Subjects Review Board of the University of Florence and were reported according to ARRIVE guidelines [17].

### 2.9. Oxaliplatin-Induced Neuropathic Pain Model and Andrographolide Administration

Neuropathy was induced in rats by the intraperitoneal (i.p.) injection of oxaliplatin (2.4 mg/kg; Carbosynth, Compton, UK), administered for 5 consecutive days every week for 2 weeks (10 administrations performed on the days 1–5 and the days 8–12) [17,18]. Oxaliplatin was dissolved in a 5% glucose solution. AG-loaded escinosomes (2 mg/mL) were diluted 1:2 in saline solution and acutely subcutaneously administered (10 mL/kg) when neuropathy was well established (day 15). Either free AG or unloaded escinosomes were suspended in saline solution, and each one was subcutaneously administered at the same dose contained in AG-loaded escinosomes.

### 2.10. Assessment of Thermal Allodynia (Cold Plate Test)

In order to evaluate thermal allodynia, animals were subjected to the Cold Plate Test (Ugo Basile, Varese, Italy) [19]. Briefly, rats were placed onto a cold surface kept at a constant temperature of 4 ± 1 °C inside a cylindrical plexiglas chamber (diameter: 10 cm, height: 15 cm) with an open top. The time (s) at which the animal showed the first pain-related behaviour (paw lifting or licking) was recorded and considered a quantitative measure of the animal’s pain threshold. The cut-off time latency was set at 30 s.

### 2.11. Irwin Test

To evaluate the safety profile of the AG-loaded escinosomes (10 mg/kg, acutely administered to oxaliplatin-treated rats), 30 min after s.c. administration, the Irwin test was performed to analyze neurobehavioral or physiological parameters. Each rat was placed in a transparent cage (26 × 41 cm^2^), and, according to Irwin, 26 parameters were systematically assessed. Behavioural, autonomic, and neurological manifestations produced by compound administration in rats were evaluated: motor displacement, motor reflexes, stereotypies, grooming, reaction to painful or environmental stimuli (analgesia, irritability), startle response, secretions, excretions, respiratory movements, skin color and temperature, piloerection, exophthalmos, eyelid and corneal reflexes, muscle tone, ataxia, tremors, head twitches, jumps, convulsions, Straub tail, and other signs or symptoms. For postural reflexes (righting reflex) and other signs such as piloerection, exophthalmia (exaggerated protrusion of the eyeball), ataxia, tremors, and Straub tail, only presence or absence was recorded. Skin color was evaluated qualitatively (pale, red, or purple); other signs were evaluated semi-quantitatively, according to the observer’s scale (0 to + 4, −4 to 0, or −4 to + 4). The terms sedation and excitation express the final interpretation of a group of signs. Sedation was characterised by reduced motor activity, reduced startle response, eyelid ptosis, and reduced response to manual manipulation. Excitation was characterised by increased motor activity, increased startle response, increased response to manual manipulation and exophthalmia. Hyperactivity includes running, jumping, and attempts to escape from the container. Trained observers not informed about the specific treatment of each animal group carried out this test.

### 2.12. Statistical Analysis

An investigator blinded to the treatments performed the behavioral assessments. The results reported the mean ± S.E.M. of 5 rats for each experimental group. Data were analyzed using the software “Origin 9” (OriginLab, Northampton, MA, USA). The analysis of variance and the post-hoc comparison were performed by one-way ANOVA and Bonferroni’s significant difference procedure, respectively. *p* values less than 0.05 or 0.01 were considered significant.

## 3. Results

### 3.1. Preparation and Light Scattering Analysis of Escinosomes

In the present study, escinosomes were evaluated for their versatility in loading a lipophilic drug, as AG, with low aqueous solubility, limiting bioavailability. The study also aimed to test the escinosome suitability for subcutaneous administration for treating oxaliplatin-induced neuropathy in mice. AG-loaded escinosomes were formulated by the thin layer evaporation method, using the same concentrations of P90G and ESN (33 mg/mL and 5mg/mL, respectively) reported in the previous study [10], and adding 2 mg/mL of AG (0.2% *w*/*v*) to the lipid phase. Size and PdI of the obtained formulation were improved by varying the volume of the hydration medium (5 mL or 10 mL), time and amplitude of sonication, equilibration time, and time and temperature of rest, as reported in Table 1.

Changes in escinosome sizes during the sonication process were visually perceived because the milky appearance of the formulation turned slightly transparent. Specifically, the preparation was optimized in 10 mL because a lower PdI was obtained by hydrating the lipid film with this volume instead of 5 mL. Generally, a PdI below 0.3 is considered acceptable in drug delivery applications and indicates a homogenous population of vesicles [20,21]. A higher sonication amplitude enabled excellent homogeneity. Thus, 48% of power was selected for the formulation optimization. The equilibration time using the magnetic stirrer at 25 ± 2 °C was fixed at 15 min because no differences were observed between samples let to stabilize 30 min or 60 min. However, a short equilibration time was settled to allow the formulation to cool down after the ultrasonication. Additionally, it was observed that the PdI decreased after storing the formulation overnight, especially at low temperatures (4 °C). The number 14 was the best formulation (Table 1), with an average diameter of 164.7 ± 13.30 nm and a PdI of 0.190 ± 0.0890 obtained from the mean of 10 different preparations. The high ζ-potential (−35.4 ± 0.451 mV) indicated good nanovesicle stability and a low tendency to aggregate. The formulation was expected to have a good performance in vivo because the sizes of nanocarriers deeply influence their absorption. After subcutaneous injection, vesicles do not have direct access to the bloodstream as the permeability of blood capillaries is restricted to water and small molecules [22,23]. In contrast, larger vesicles up to about 150 nm are easily absorbed by lymphatic capillaries draining the subcutaneous injection site. Vesicles over a few hundred nanometers in size will be trapped in the interstitial space for an extended period and will slowly destabilize and degrade in time, concurrently losing their drug content, or they will be transported by dendritic cells [22,23]. Escinosomes developed in the present study had suitable sizes to reach the systemic circulation by the lymphatic capillaries and to release the drug in the injection site.

### 3.2. Physical Characterization of Escinosomes by Cryo Transmission Electron Microscopy

**Figure 3:**
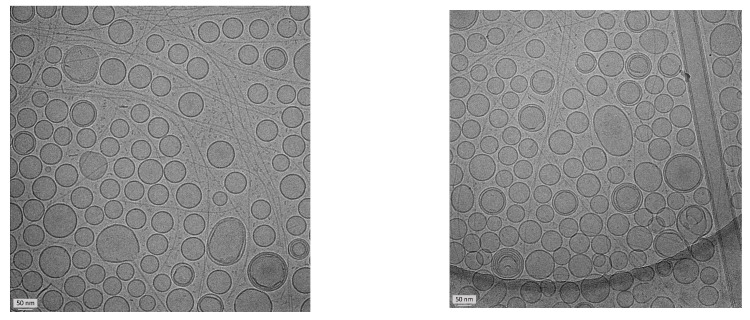
Pictures of AG-loaded escinosomes obtained by Cryo-TEM. Scale bar = 50 nm.

The morphology and architecture of AG-loaded escinosomes were evaluated using the Cryo-TEM. Nanovesicles had a delineated spherical shape, sometimes showing a bilamellar or oligolamellar structure (Figure 3). In addition, the average dimensions were consistent with those obtained by DLS analysis.

### 3.3. Chemical Characterization of Escinosomes

Chemical characterization of AG-loaded escinosomes was carried out by determining R% and EE% of ESN and AG. ESN was also evaluated because it is both a component of the vesicle bilayer and an active ingredient and possibly contributes to the efficacy of the treatment. The resulting values of R% were very high for both ESN and AG (Table 2). AG was significantly loaded in the escinosomes (EE% around 88%), and the excellent ESN encapsulation (about 97%) confirmed that the saponin was a vesicle bilayer component and strongly interacted with phosphatidylcholine molecules.

The selection of the time and conditions to purify vesicles from the not encapsulated or superficially adsorbed drug, avoiding the release of the same drug, is critical in evaluating EE%. To validate the selected time (1 h), dialysis with the physical mixture of ESN and AG in 10% *v*/*v* Tween20/PBS was performed [5]. After 1 h, about 20% of ESN and 50% of AG had diffused in the dialysis medium. Since both these percentages are higher than those calculated by difference for the formulation’s dialysis, 1 h can be considered a suitable time to evaluate the EE% of both molecules without overestimation.

### 3.4. Stability Study of Escinosomes

**Figure 4:**
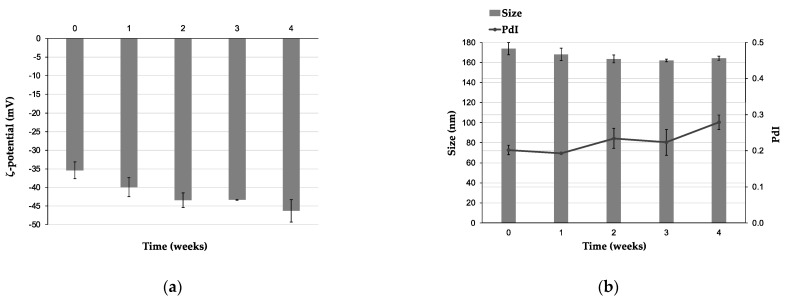
Physical stability of AG-loaded escinosomes regarding (**a**) ζ-potential, and (**b**) size and PdI of nanovesicles. Data are expressed as Mean ± SD (*n* = 3).

**Figure 5:**
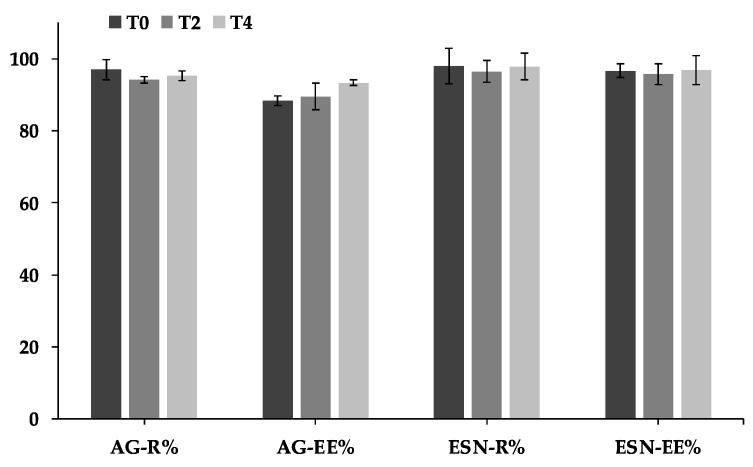
Chemical stability of AG-loaded escinosomes regarding recovery (R%) and encapsulation efficiency (EE%) of escin (ESN) and andrographolide (AG) measured at T_0_ (time zero), T_2_ (after 2 weeks), and T_4_ (after 4 weeks). Data are expressed as Mean ± SD (*n* = 3).

AG-loaded escinosomes were stored at 4 °C for 4 weeks to evaluate their physical and chemical stability. Size, PdI, and ζ-potential were monitored every 7 days by DLS and ELS. The ζ-potential gradually increased over 4 weeks, illustrating a great repulsion between nanovesicles and good physical stability to aggregation (Figure 4a). In addition, the average diameter remained constant over time (Figure 4b). A slight increase of PdI was observed after 2 and 4 weeks of storage, but the value remained below 0.3 (Figure 4b). A PdI below 0.3 is considered acceptable in drug delivery applications and indicates a homogenous population of vesicles [21]. R% and EE% of the two active substances, assessed every 2 weeks by HPLC-DAD analysis, were also constant for all the monitored time, as displayed in the graph in Figure 5. Therefore, AG-loaded escinosomes were chemically stable for 1 month of storage at 4 °C, far from the light, and, considering the slight increase of PdI, physically stable for at least 3 weeks.

### 3.5. Selection of the Acceptor Medium and Release Study

Solubility of ESN and AG in different buffer solutions was evaluated to select the acceptor medium for the release study. Specifically, the solubility of the active substances was tested in PBS, 1% *v*/*v* Tween 20/PBS, and 5% *v*/*v* Tween 20/PBS at 25 ± 2 °C for 24 h, maintaining the suspensions under magnetic stirring. ESN solubility was found to be higher than 5 mg/mL for the two solutions with Tween 20. PBS with 5% *v*/*v* Tween 20 was finally selected as acceptor medium because the ratio between solubility and AG maximum concentration achievable in the acceptor compartment (S/C ratio) was around 11, while it was around 5 for 1% *v*/*v* Tween 20/PBS and 3 for PBS (Table 3). A ratio higher than 10 is recommended to maintain sink conditions for the whole of the experiment time without affecting drug passive diffusion across the semi-permeable membrane.

The release rate of AG and ESN from escinosomes was evaluated using the dialysis bag method [15,16]. A gradual and prolonged AG release from escinosomes was observed, without burst effects typical of conventional dosage forms. These findings evidence the advantage of escinosomes as an innovative drug delivery system with controlled release properties. The released AG after 24 h was 85.3 ± 1.36% (mean ± SD; *n* = 3). By contrast, ESN was not detected during the experiment because the concentration was always under the limit of detection [10], confirming that escinosomes remained physically and chemically stable for the whole time of the experiment. ESN, indeed, was supposed to be a vesicle bilayer component, and the low release from vesicles proves this interaction.

### 3.6. In Vivo Evaluation of AG-Loaded Escinosomes Efficacy against Neuropathic Pain

**Figure 6:**
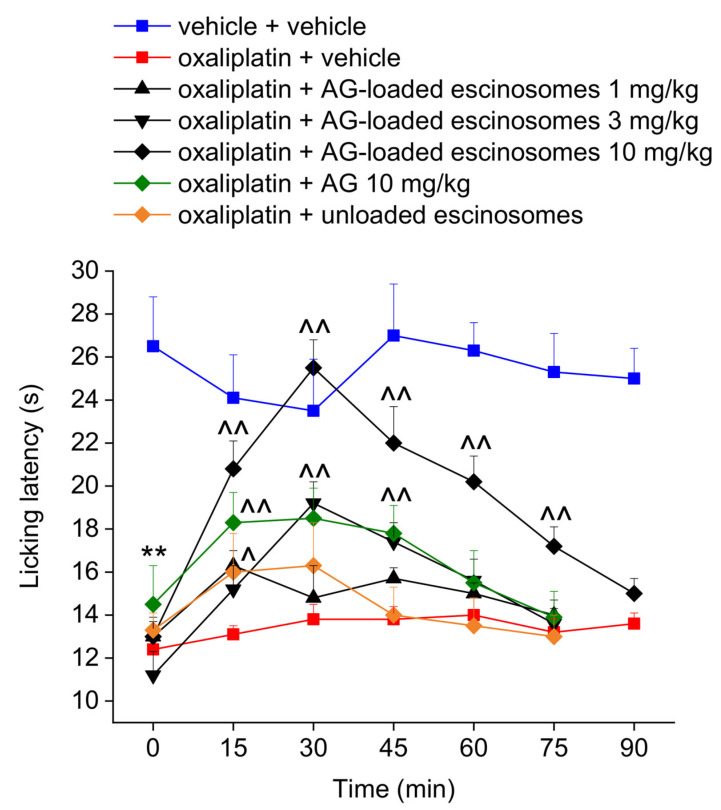
Effect of acute administration of AG-loaded escinosomes on oxaliplatin-induced neuropathic pain in rats. AG-loaded escinosomes were diluted in saline solution and acutely subcutaneously administered when the neuropathy was well established (day 15). Both AG and unloaded escinosomes were suspended in saline solution and subcutaneously administered at the same dose contained in AG-loaded escinosomes. Each value represents the Means ± SEM of 5 rats per group. Statistical analysis was one-way ANOVA followed by Bonferroni’s post-hoc comparison. ** *p* < 0.01 vs. vehicle + vehicle-treated animal; ^ *p* < 0.05 and ^^ *p* < 0.01 vs. oxaliplatin + vehicle-treated animals.

Figure 6 shows the effect of subcutaneous administration of AG-loaded escinosomes on oxaliplatin-induced thermal allodynia in rats. The cold plate test was performed when neuropathic pain was well-established in rats. On day 15, oxaliplatin-treated rats showed a licking latency (12.4 ± 0.2 s), significantly lower than controls (23.5 ± 2.3 s) (Figure 6). A single subcutaneous administration of AG-loaded escinosomes relieved thermal allodynia dose-dependently, as depicted in Figure 6. The subcutaneous administration of the higher dose of AG-loaded escinosomes (equivalent to 10 mg/kg formulated AG) completely counteracted thermal allodynia caused by oxaliplatin treatment. This effect started 15 min after injection and lasted up to 75 min. The dose 3 mg/kg of formulated AG was partially effective, resulting in a significant increase of animals’ licking latency time only between 30 min and 45 min. The lower dose of formulated AG (1 mg/kg) resulted in ineffectiveness. Although subcutaneously administering free AG (10 mg/kg) suspended in saline solution led to a significant increase of animals’ pain threshold between 15 and 45 min after the injection, it resulted in being less effective than the equivalent dose of AG loaded in escinosomes. On the other hand, administering the same amount of unloaded escinosomes did not elicit any significant effect on pain (Figure 6). The safety profile of the formulation AG-loaded escinosomes (10 mg/kg) was tested 30 min after the administration to oxaliplatin-treated rats by the Irwin test. None-behavioral, autonomic, or neurological alteration was observed (Table S1 from Supplementary Material).

## 4. Discussion

In this study, escinosomes, novel nanovesicles consisting of phosphatidylcholine and ESN, were loaded with AG and characterized for their physical, chemical and technological properties. AG-loaded escinosomes were then administered acutely subcutaneously in a murine model of neuropathy induced by oxaliplatin.

AG has anti-inflammatory, anti-edema, and anti-nociceptive activities and it represents a valuable natural complement in treating painful musculoskeletal diseases, like arthritis [24,25,26,27] endometriosis, and colitis [28,29,30]. This natural compound has also been reported to relieve pain in animal models of neuropathic pain induced by HIV or spared nerve injury [31,32]. However, until now, AG has not reached its therapeutic potential due to its low aqueous solubility and scarce bioavailability when administered by oral conventional dosage forms.

Escinosomes are special nanovesicles where ESN is involved in the bilayer structure, imparting physical stability and high deformability to the vesicles, and allowing controlled drug release and improved penetration across the skin as previously reported [10]. The present study investigated the escinosome versatility in loading other drugs and their suitability for the subcutaneous administration route. AG was a perfect candidate drug because of the low aqueous solubility, limiting its bioavailability. The success of developed AG-loaded escinosomes is primarily due to the biocompatibility, high loading capacity of lipophilic active molecules, and the controlled release properties of the nanocarrier. Developed AG-loaded escinosomes had an average diameter of about 165 nm, PdI of 0.190, and ζ-potential of ca. −35 mV, indicating optimal size homogeneity and high repulsion between nanovesicles, signifying stability and low tendency to aggregate. This property is critical for the good storage of the formulation. Physical stability studies revealed only a slight increase in PdI during one month of storage, but the sizes remained constant, and ζ-potential positively increased in absolute value. In addition, the escinosome loading with the active substance was unchanged over time (ca. 88% for AG and 97% for ESN). Morphologic analysis was performed by Cryo-TEM, revealing numerous and clear nanovesicles, mainly with unilamellar structures and dimensions consistent with DLS results. Escinosomes allowed a prolonged release of AG over time (about 85% after 24 h), while ESN was not released, confirming the stability of the vesicle structure at 37 °C.

This study represents the first investigation exploring the efficacy of AG against chemotherapy-induced neuropathic pain, which still represents an unsolved therapeutic problem in many patients. In vivo data demonstrated that AG anti-hyperalgesic effect was significantly enhanced and prolonged by employing escinosomes as a delivery system. Therefore, this formulation might overcome bioavailability issues occurring with AG-based conventional preparations [33], avoiding therapeutic failures. Finally, AG was reported to enhance the efficacy of anti-cancer treatments [34,35]. This evidence contributes to its safety on the employment in patients undergoing chemotherapy.

## Figures and Tables

**Table 1:** Preparation of AG-loaded escinosomes and analysis of Size and PdI. Results are expressed as Mean ± SD (at least *n* = 3).

Formulation	Hydration Volume (mL)	Sonication Time and Amplitude (min -%)	Equilibration Time at 25 °C (min)	Temperature of 1-Night Storage (°C)	Size (nm)	PdI
1	5	0	0	NS	342.4 ± 11.80	0.377 ± 0.0531
2	5	5–48	30	NS	142.4 ± 0.1381	0.426 ± 0.0217
3	5	5–48	60	NS	135.1 ± 0.6249	0.416 ± 0.00320
4	5	5–48	60	4	112.2 ± 2.185	0.362 ± 0.0110
5	5	5–48	60	25	136.5 ± 3.510	0.363 ± 0.0192
6	5	10–48	30	NS	101.4 ± 12.72	0.500 ± 0.0586
7	5	10–48	60	NS	120.9 ± 13.91	0.608 ± 0.0651
8	5	10–48	60	4	122.9 ± 13.72	0.526 ± 0.116
9	5	10–48	60	25	142.4 ± 11.32	0.589 ± 0.0720
10	10	0	0	NS	761.4 ± 39.81	0.545 ± 0.0913
11	10	1–30	15	NS	259.3 ± 3.574	0.451 ± 0.0187
12	10	5–30	15	NS	111.3 ± 3.156	0.421 ± 0.0123
13	10	10–30	15	NS	88.10 ± 0.921	0.387 ± 0.0130
14	10	10–48	15	4	164.7 ± 13.31	0.190 ± 0.0891

NS = no storage.

**Table 2:** Recovery (R%) and encapsulation efficiency (EE%) of escin (ESN) and andrographolide (AG) in the escinosomes. Data are expressed as Mean ± SD (*n* = 3).

	R%	EE%
ESN	98.4 ± 4.63	96.7 ± 1.98
AG	97.0 ± 2.81	88.3 ± 1.43

**Table 3:** Solubility and solubility/maximum concentration ratio (S/C) of andrographolide (AG) in different buffer solutions. Data are showed as Mean ± SD (*n* = 3).

Acceptor Medium	AG Solubility (mg/mL)	S/C Ratio
PBS	0.0582 ± 0.000805	3
1% *v*/*v* Tween 20/PBS	0.0912 ± 0.00967	5
5% *v*/*v* Tween 20/PBS	0.213 ± 0.000782	11

## Data Availability

The data presented in this study are available on request from the corresponding author. The data are not publicly available due to privacy restrictions.

## Supplementary Materials

The following supporting information can be downloaded at: https://www.mdpi.com/1999-4923/14/3/493/s1, Table S1. Effect of the acute administration of AG-loaded escinosomes on oxaliplatin-induced behavioral, autonomic, and neurological alterations by Irwin test. The Irwin test was performed 30 min following the acute administration of the treatments in oxaliplatin-treated rats. The signs were evaluated semi-quantitatively, according to the observer’s personal scale (0 to +4, −4 to 0, or −4 to +4). Each value represents the mean ± SEM of 5 rats per group. * *p* < 0.05 vs. vehicle + vehicle group.

## Sample Availability

Samples of the pure constituents and formulations are available from the authors.
