# Supplementary material for: Escinosomes: Safe and Successful Nanovesicles to Deliver Andrographolide by a Subcutaneous Route in a Mice Model of Oxaliplatin-Induced Neuropathy
Source: Pharmaceutics. 2022 Feb 24;14(3):493. doi: 10.3390/pharmaceutics14030493 (PMC8949339; doi:10.3390/pharmaceutics14030493)
Supplement: Supplementary file 1 [file pharmaceutics-14-00493-s001.zip › pharmaceutics-1555293-supplementary.pdf]

pharmaceutics

MDPI

Supplementary Material

Escinosomes: Safe and Successful Nanovesicles to Deliver Andrographolide by a Subcutaneous Route in a Mice Model of Oxaliplatin-Induced Neuropathy

Giulia Vanti [1], Michela Capizzi [1], Lorenzo Di Cesare Mannelli [2], Elena Lucarini [2], Maria Camilla Bergonzi [1], Carla Ghelardini [2] and Anna Rita Bilia [1,*]

[1] Department of Chemistry, University of Florence, Via Ugo Schiff 6, 50019, Florence, Italy; giulia.vanti@unifi.it (G.V.); michela.capizzi@stud.unifi.it (M.C.); mc.bergonzi@unifi.it (M.C.B.)
[2] Pharmacology and Toxicology Section, Department of Neuroscience, Psychology, Drug Research and Child Health (NEUROFARBA), University of Florence, Viale G. Pieraccini 6, 50139 Firenze, Italy; lorenzo.mannelli@unifi.it (L.D.C.M.); elena.lucarini@unifi.it (E.L.); carla.ghelardini@unifi.it (C.G.)
* Correspondence: ar.bilia@unifi.it; Tel.: +39-0554-573709

Table S1. Effect of the acute administration of AG-loaded escinosomes on oxaliplatin-induced behavioral, autonomic, and neurological alterations by Irwin test. The Irwin test was performed 30 minutes following the acute administration of the treatments in oxaliplatin-treated rats. The signs were evaluated semi-quantitatively, according to the observer's personal scale (0 to + 4, − 4 to 0, or − 4 to + 4). Each value represents the mean ± SEM of 5 rats per group. **p* < 0.05 vs vehicle + vehicle group.

	Treatments					
	**Vehicle + Vehicle**	**OX + Vehicle**	**OX + AG-Esc 10 mg/kg**	**OX + AG 10 mg/kg**	**OX + Esc**	**Limits**
**Behavior**						
Spontaneous activity	4 ± 0	2.3 ± 0.2*	2.1 ± 0.1	2.4 ± 0.2	2.0 ± 0.2	4–0
Passivity	0 ± 0	2.0 ± 0.1*	1.9 ± 0.2	2.0 ± 0.2	2.2 ± 0.1	0–4
Cleaning	4 ± 0	4 ± 0	4 ± 0	4 ± 0	4 ± 0	4–0
Curiosity	4 ± 0	2.5 ± 0.2*	2.3 ± 0.1	2.3 ± 0.1	2.6 ± 0.2	4–0
Reactivity	4 ± 0	2.2 ± 0.1*	2.0 ± 0.2	2.3 ± 0.1	2.4 ± 0.1	4–0
Vocalization	0 ± 0	0 ± 0	0 ± 0	0 ± 0	0 ± 0	0–4
**Central Nervous System Excitement**						
Straub tail	0 ± 0	0 ± 0	0 ± 0	0 ± 0	0 ± 0	0–4
Tremors	0 ± 0	0 ± 0	0 ± 0	0 ± 0	0 ± 0	0–4
Convulsions	0 ± 0	0 ± 0	0 ± 0	0 ± 0	0 ± 0	4–0
**Movement**						
Ataxia	0 ± 0	0 ± 0	0 ± 0	0 ± 0	0 ± 0	0–4
Stereotypies	0 ± 0	0 ± 0	0 ± 0	0 ± 0	0 ± 0	0–4
Straightening reflex	4 ± 0	4 ± 0	4 ± 0	4 ± 0	4 ± 0	4–0
**Muscular Tone**						
Physical strength	4 ± 0	4 ± 0	4 ± 0	4 ± 0	4 ± 0	4–0
Palpebral reflex	4 ± 0	4 ± 0	4 ± 0	4 ± 0	4 ± 0	4–0
**Autonomic Signs**						
Piloerection	0 ± 0	2.6 ± 0.1*	2.7 ± 0.1	2.5 ± 0.1	2.5 ± 0.2	0–4
Exophthalmos	0 ± 0	0 ± 0	0 ± 0	0 ± 0	0 ± 0	0–4
Cyanosis	0 ± 0	0 ± 0	0 ± 0	0 ± 0	0 ± 0	0–4
Flush	0 ± 0	0 ± 0	0 ± 0	0 ± 0	0 ± 0	0–4
Pallor	0 ± 0	2.8 ± 0.1*	2.7 ± 0.2	3 ± 0.1	2.7 ± 0.1	0–4
Palpebral opening	4 ± 0	4 ± 0	4 ± 0	4 ± 0	4 ± 0	4–0

Salivation	0 ± 0	0 ± 0	0 ± 0	0 ± 0	0 ± 0	0–4
Lacrimation	0 ± 0	0 ± 0	0 ± 0	0 ± 0	0 ± 0	0–4
Hypohyperthermia	0 ± 0	0 ± 0	0 ± 0	0 ± 0	0 ± 0	− 4/+ 4
**Toxicity**						
Immediate death	0 ± 0	0 ± 0	0 ± 0	0 ± 0	0 ± 0	0–4
Delayed death (48 h)	0 ± 0	0 ± 0	0 ± 0	0 ± 0	0 ± 0	0–4

OX = oxaliplatin; AG = andrographolide; Esc = unloaded escinosomes; AG-Esc = AG-loaded escinosomes
